# Cellular Immunity of SARS-CoV-2 in the Borriana COVID-19 Cohort: A Nested Case–Control Study

**DOI:** 10.3390/epidemiologia5020012

**Published:** 2024-04-10

**Authors:** Salvador Domènech-Montoliu, Joan Puig-Barberà, María Rosario Pac-Sa, Alejandro Orrico-Sanchéz, Lorna Gómez-Lanas, Diego Sala-Trull, Carmen Domènech-Leon, Alba Del Rio-González, Manuel Sánchez-Urbano, Paloma Satorres-Martinez, Laura Aparisi-Esteve, Gema Badenes-Marques, Roser Blasco-Gari, Juan Casanova-Suarez, María Gil-Fortuño, Noelia Hernández-Pérez, David Jovani-Sales, Laura López-Diago, Cristina Notari-Rodríguez, Oscar Pérez-Olaso, María Angeles Romeu-Garcia, Raquel Ruíz-Puig, Alberto Arnedo-Pena

**Affiliations:** 1 Medical Direction University Hospital de la Plana, 12540 Vila-real, Spain; pttcarmen@hotmail.com; 2 Vaccines Research Unit, Foundation for the Promotion of Health and Biomedical Research in Valencia Region FISABIO-Public Health, 46020 Valencia, Spain; jpuigb55@gmail.com (J.P.-B.); alejandro.orrico@fisabio.es (A.O.-S.); 3 Public Health Center, 12003 Castelló de la Plana, Spain; charopac@gmail.com (M.R.P.-S.); aromeu96@gmail.com (M.A.R.-G.); 4 Epidemiology and Public Health (CIBERESP), 28029 Madrid, Spain; 5 Secretary of Chair of Vaccines Catholic University of Valencia, 46001 Valencia, Spain; 6 Emergency Service University Hospital de la Plana, 12540 Vila-real, Spain; lornagl78@gmail.com (L.G.-L.); saladiego2@gmail.com (D.S.-T.); manu.msu@gmail.com (M.S.-U.); palomasatmar@gmail.com (P.S.-M.); gemabamar@hotmail.com (G.B.-M.); roserblascog@gmail.com (R.B.-G.); jovasal1987@gmail.com (D.J.-S.); notari_cri@gva.es (C.N.-R.); raquelruizpuig@gmail.com (R.R.-P.); 7 Department of Medicine, University CEU Cardenal Herrera, 12006 Castelló de la Plana, Spain; carmendomenech04@gmail.com; 8 Health Centers I and II, 12530 Borriana, Spain; delrio_alb@gva.es; 9 Carinyena Health Center, 12540 Vila-real, Spain; lauraaparisiesteve@gmail.com; 10 Nursing Service University Hospital de la Plana, 12540 Vila-real, Spain; juancasanova83@gmail.com; 11 Microbiology Service University Hospital de la Plana, 12540 Vila-real, Spain; gil_marfor@gva.es (M.G.-F.); hernandez_noeper@gva.es (N.H.-P.); perez_oscola@gva.es (O.P.-O.); 12 Clinical Analysis Service University Hospital de la Plana, 12540 Vila-real, Spain; lopez_laudia@gva.es; 13 Department of Health Science, Public University Navarra, 31006 Pamplona, Spain

**Keywords:** COVID-19, SARS-CoV-2 infection, cellular immunity, T cells, CD4+, CD8+, humoral immunity, anti-S antibodies, anti-N antibodies, nested case–control, associated factors

## Abstract

Our goal was to determine the cellular immune response (CIR) in a sample of the Borriana COVID-19 cohort (Spain) to identify associated factors and their relationship with infection, reinfection and sequelae. We conducted a nested case–control study using a randomly selected sample of 225 individuals aged 18 and older, including 36 individuals naïve to the SARS-CoV-2 infection and 189 infected patients. We employed flow-cytometry–based immunoassays for intracellular cytokine staining, using Wuhan and BA.2 antigens, and chemiluminescence microparticle immunoassay to detect SARS-CoV-2 antibodies. Logistic regression models were applied. A total of 215 (95.6%) participants exhibited T-cell response (TCR) to at least one antigen. Positive responses of CD4+ and CD8+ T cells were 89.8% and 85.3%, respectively. No difference in CIR was found between naïve and infected patients. Patients who experienced sequelae exhibited a higher CIR than those without. A positive correlation was observed between TCR and anti-spike IgG levels. Factors positively associated with the TCR included blood group A, number of SARS-CoV-2 vaccine doses received, and anti-N IgM; factors inversely related were the time elapsed since the last vaccine dose or infection, and blood group B. These findings contribute valuable insights into the nuanced immune landscape shaped by SARS-CoV-2 infection and vaccination.

## 1. Introduction

Three years have passed since the SARS-CoV-2 pandemic began, leading to roughly 6.9 million fatalities, globally. Over half of the world’s population has been infected, with a significant number of those suffering long-term sequelae, and yet the virus persists as a threat [1]. Research on the SARS-CoV-2 evolution, the pandemic’s progression and its impact on human health remains critical; this includes studying the immune response to the infection and vaccinations. Such research is essential for guiding health strategies and medical interventions [2,3].

The immune response to SARS-CoV-2 infection is characterized by humoral and cellular immunity [4]. Research indicates that specific CD4+ and CD8+ T cells are linked to less severe forms of the disease [5,6,7]. SARS-CoV-2 vaccines effectively reduce the severity and hospitalization rates caused by the virus, but they do not prevent infection nor stop transmission. The vaccine’s protection against severe outcomes is largely attributed to a cellular immune response [8,9]. The study of cellular immunity is crucial for understanding protection against SARS-CoV-2, its emerging variants and for the development of new vaccines [10,11]. Testing cellular immunity protection is challenging due to the complex, laborious and specialized techniques required to understand the biological mechanisms involved [12].

The research on the cellular immune response to SARS-CoV-2 infection has focused mainly on patients with severe outcomes, those hospitalized or suffering from long SARS-CoV-2 infection and immune-compromised individuals, with less emphasis on other populations [13]. Our investigation began with the SARS-CoV-2 outbreak at the Falles Festival in March 2020, with 570 cases among 1332 attendees at Borriana, Valencia Community (Spain); since then, we have conducted several of follow-up studies [14,15,16]. In the latest follow-up, we analyzed a randomly selected sample from the Borriana COVID-19 cohort. This sample mixed individuals who were either naïve to SARS-CoV-2 infection or had experienced reinfection. We aimed to determine the dynamics between SARS-CoV-2 infection, its clinical outcomes, and the associated cellular and humoral immune responses. We also sought to understand the effects of SARS-CoV-2 vaccination and other relevant factors in this population.

## 2. Materials and Methods

### 2.1. Study Cohort

This cohort has been previously studied three times. The first study was conducted in May 2020 with 1332 participants [14]. The second study was conducted in October 2020, evaluating only SARS-CoV-2–infected patients and including 484 participants [17]. The third study was conducted in June 2022 and included 722 participants who had undergone at least one laboratory test to verify their SARS-CoV-2 infection status [18].

In December 2022, we conducted a nested case–control study on a randomly selected sample of 225 subjects aged 18 years or older from the third study based on the following criteria: (1) a 1:4 ratio of naïve (never infected) participants versus SARS-CoV-2 infection cases, and (2) a difference of 13% in cellular immune response between the two groups, with a power of 80% and alpha error of 5%, following research comparing naïve and SARS-CoV-2–infected patients [19,20,21]. The final theoretical sample included 45 naïve participants and 180 SARS-CoV-2–infected patients. In addition, we estimated from our previous data that 33% of the SARS-CoV-2–infected patients experienced post–COVID-19 sequelae, showing a 20% chance of SARS-CoV-2 reinfection in the third study [22]. A sample size of 103 was calculated for participants with sequelae and 89 for participants with reinfection, based on a precision of 5% and power of 90%. These estimations were made with Stata ^®^14 version 2 (Stata Corp, College Station, TX, USA). and OpenEpi (https://www.openepi.com) programs (accessed on 20 September 2022)

The study groups were divided into four categories: (1) naïve participants, (2) SARS-CoV-2–infected patients, (3) SARS-CoV-2–infected patients with sequelae, and (4) patients with SARS-CoV-2 reinfections or no reinfections.

In all 225 subjects, we obtained blood samples to perform the following determinations:

Anti–SARS-CoV-2 spike (S) IgG, nucleocapsid (N) IgG, and nucleocapsid (N) IgM antibodies were estimated by chemiluminescence microparticle immunoassay (CMIA) (Alinity I Abbott Laboratories, Chicago, USA) [23]. All these tests were performed at the Microbiology Service Laboratory of La Plana University Hospital, Vila-real (Spain).

Cellular immunity against the Wuhan and Omicron BA.2 variants of concern (VOC) were measured using flow cytometry. Functional cellular assays were based on the detection of T-cell–activation markers. Enumeration of SARS-CoV-2-S–reactive interferon-γ–producing CD4+ and CD8+ T cells in fresh heparinized peripheral whole blood was carried out by flow cytometry immunoassay for intracellular cytokine staining (BD Fastimmune, Becton Dickinson and Company-Biosciences, San Jose, CA, USA,) as previously described [21,24,25,26]. Specimens were analyzed at the Microbiology Service, Clinic University Hospital, INCLIVA Health Research Institute, Valencia, (Spain).

Levels of 25-hydroxy vitamin D [25 (OH) D] were measured by electrochemiluminescence-based assay (Elecsys vitamin D total II Roche Diagnostic, Germany) [27]. This analysis was performed at the Clinical Laboratory Service of La Plana University Hospital, Vila-real (Spain).

In June 2022, we ran a questionnaire in collaboration with the health staff of La Plana Hospital, Castellon Public Health Center, and the health centers of Borriana, Vila-real, Onda, and La Vall d’Uixò. The interviews were conducted by phone or face-to-face and we collected information about demographic characteristics, chronic diseases, health habits, body mass index (BMI) (kg/m^2^), occupation, level of physical exercise, smoking habits, alcohol consumption, chronic illnesses and SARS-CoV-2 infection exposures, including laboratory-confirmed SARS-CoV-2 infection and reinfection, and SARS-CoV-2 infection sequelae. To obtain information about SARS-CoV-2 vaccination, we consulted the vaccination records of the Valencia Community for data on administration date, vaccine type and brand. Finally, those who were vaccinated were asked about vaccine-derived adverse effects. Subsequently, in December 2022, at the time of the blood sampling, we ran an additional short questionnaire to ascertain rates of SARS-CoV-2 infection, reinfection and sequelae.

We defined reinfection as a new SARS-CoV-2 infection more than 60 days after the previous SARS-CoV-2 infection and confirmed by polymerase chain reaction test (PCR) or a rapid antigen test (RAT) [28]. The first infection must have been confirmed by either PCR, RAT, or positive anti-nucleocapsid IgG determination. We considered a subject to have suffered post–SARS-CoV-2 infection sequelae if they provided a positive response to the question “Do you have some attributable sequelae by COVID-19 disease?” on the questionnaire administered in June 2022.

### 2.2. Statistical Methods

We employed the following descriptive statistics to explain our results: mean, standard deviation, median, and ranges. To compare qualitative data, we applied Chi-square and Fisher’s exact tests. For quantitative data, we used the Kruskal–Wallis and median tests. Spearman’s correlation non-parametric coefficient (rs) was used to analyze the strength and direction of the association between anti-S IgG levels and cellular immunity across the study groups. The rs coefficient, which ranges from −1 to +1, indicates a perfect positive or negative rank correlation at its extremes, while zero denotes no correlation.

The nested case–control comprised of three separate studies: (1) SARS-CoV-2–infected patients (cases) versus naïve participants (controls); (2) SARS-CoV-2–infected patients with sequelae (cases) versus patients without sequelae (controls); (3) SARS-CoV-2 patients with reinfection (cases) versus no reinfection (controls).

The dependent variable was the cellular immune response as measured by the production of CD4+ and CD8+ T cells upon stimulation with Wuhan and BA.2 (Omicron) antigens. We defined a positive response as any percentage of response above zero, contrasting with a zero-percentage indicating a negative response. Accordingly, we ran three nested case–control studies to assess the impact on the immune response considering the different experiences in the subjects of our sample across the three groups explained above. We calculated odds ratios (OR) with a 95% confidence interval (95% CI) to compare cases and controls. The total sample population was used to study potential factors associated with the cellular immune response.

We identified potential confounders such as age, sex, time elapsed since SARS-CoV-2 infection or reinfection, or since the last SARS-CoV-2 vaccine dose, BMI, smoking status, alcohol consumption, habitual physical exercise, chronic diseases, and number of anti-SARS-CoV-2 vaccine doses received, using Directed Acyclic Graphics (DAGs) [29]. Time elapsed since SARS-CoV-2 infection or reinfection, or since the last SARS-CoV-2 vaccine dose was received were considered as the time elapsed since these events could play a role in the immune response. We employed multivariable logistic regression models for confounder adjustment. We conducted our statistical analyses using the program Stata^®^ 14 version 2.

To ensure the robustness of our finding, we conducted a sensitivity analysis. This analysis was based on a definition of a positive immune response of ≥ 0.10% for CD4+ and CD8+ T cells, following the methodology outlined by Gimenez and co-authors [24]. This approach allowed us to confirm the consistency of our results under varying criteria for immune response.

This study was approved by the Ethics Committee of the La Plana University Hospital. All participants provided written informed consent.

## 3. Results

From the initial pool of 619 participants aged 18 years and older, we obtained a random sample of 225 individuals. This sample was categorized based on SARS-CoV-2 infection status. Nine individuals were infected for the first time between June and December 2022, and the final sample included 36 naïve to SARS-CoV-2 infection, and 189 previously-infected. Within the group of previously-infected participants, we distinguished four non-exclusive subgroups: those who developed sequelae following the infection (n = 77) and those who did not (n = 103), along with participants who experienced reinfections (n = 78) and those with a single infection event (n = 88). In the previously-infected group, details on sequelae were missing for 9 individuals and 23 cases of reinfection were not laboratory-confirmed.

Characteristics of the studied population and the comparison between groups are detailed in Table 1. The average age varied across groups, with the naïve group showing a median age of 51 years and the non-sequelae group, 42 years. The naïve group was older than the SARS-CoV-2–infected patients (*p* = 0.010), and the sequelae group was older than the non-sequelae group (*p* = 0.006). Female participation was more prevalent than male in all groups, ranging from 55.6% in the naïve group to 64.9% in the SARS-CoV-2 patients with sequelae group.

The time elapsed since the last vaccine dose or infection showed minor variations in range with a median of 11 months for all groups. BMI remained fairly consistent across all groups, ranging from 25.4 kg/m^2^ to 26.4 kg/m^2^. Smoking habits were significantly higher in the naïve group compared with the infected patients at 44.4% versus 28.2% (*p* = 0.001).

Alcohol consumption showed minimal variation among the groups. Physical exercise was more frequent in patients without sequelae (64.1%) and least common in the naïve group (50%). The prevalence of chronic diseases was higher in patients with sequelae (44.2%) compared to those without sequelae (33.0%)

Vaccination rates varied across the groups. A total of 96.8% of infected patients had received at least one dose of a SARS-CoV-2 vaccine. In contrast, 100% of the naïve group had received three vaccine doses compared to 77.8% in the infected group (*p* = 0.001). The patients with sequelae had received more SARS-CoV-2 vaccine doses than the patients without sequelae (*p* = 0.034). However, the reinfection group had received less vaccine doses than the no-reinfection group (*p* = 0.028).

Anti-S IgG levels were highest in the no-reinfection group and lowest in the reinfection group (*p* = 0.005). The reinfection group also exhibited higher positivity for anti-N IgM and IgG compared to the no-reinfection group (*p* = 0.003). Positivity for anti-N IgG was observed in 56.6% of infected patients.

Vitamin D levels were highest in the sequelae group and lowest in the naïve group. Regarding ABO blood groups, O was most frequent in the naïve group and least frequent in the sequelae group. The A group was more prevalent in the no-reinfection group and least frequent in the reinfection group. The B group showed a higher prevalence in the sequelae group and lower in the group without sequelae. The AB group was most common among the naïve participants.

The distribution of the cellular immune response is shown in Table 2. The median frequency of positivity against Wuhan antigens showed differences between groups: the immune response of CD8+ T cells varied from 0.11% (0–13.5%) in the no-reinfection group to 0.07% (0–6.9%) in reinfection group; CD4+ T cells varied from 0.10% (0–1.05%) in the naïve group to 0.07% (0–1.47%) in the reinfection group. Against BA.2 antigens, the immune response of CD8+ T cells varied from 0.11% (0–6.5%) in the naïve group to 0.07% (0–13.3%) in patients without sequelae; CD4+ T cells varied from 0.13% (0–0.85%) in the naïve group to 0.07% (0–1.67%) in patients without sequelae.

Comparing percentages of positive immune responses by group, the naïve group showed a higher response for CD4+ and CD8+ T cells than infected patients. In addition, the sequelae group showed a more elevated immune response than the non-sequelae group. In contrast, the SARS-CoV-2 reinfection and no-reinfection groups presented similar immune response.

In total, 215 (95.6%) participants presented a positive cellular immune response to at least one antigen, and 10 participants (4.4%) had no response. CD4+ T cell immune response against Wuhan and BA.2 antigens was 79.2% and 77.8%, respectively. CD8+ T cells responsiveness against Wuhan and BA.2 antigens was 73.3% and 70.2%, respectively. When comparing naïve and infected patients, the naïve group had higher immunity responses, although these differences were not significant. In total, 202 participants (89.8%) showed a CD4+ T cell response and 192 participants (85.3%) showed a CD8+ T cell response.

The comparison of cellular immune responses across the studied groups are shown by a crude analysis in Table 3. The SARS-CoV-2–infected group showed a lower, but not significant, immune response than the naïve group for Wuhan and BA.2 antigens. The SARS-CoV-2 patients with sequelae showed significantly higher immune responses against Wuhan and BA.2 antigens compared to patients without sequelae, as indicated by CD4+ T cells (OR = 3.71, 95% CI 1.60–8.64) and CD4+ T cells (OR = 3.20, 95% CI 1.51–7.31), respectively. However, immune responses against Wuhan and BA.2 antigens by CD8+ T cells were, respectively, marginal or not significantly different. Immune responses of SARS-CoV-2–reinfection patients compared with non-reinfection patients were not significant.

The cellular immune response comparisons using an adjusted analysis are shown in Table 4. The SARS-CoV-2–infected patients showed lower immune responses than the naïve group to Wuhan and BA.2 antigens, except Wuhan CD8+ T cells, but again, differences were not significant. The patients with sequelae showed significantly higher immune responses than the non-sequelae group against Wuhan antigen, CD4+ T cells (OR = 3.90, 95% CI 1.50–9.52), CD8+ T cells (OR = 2.33, 95% CI 1.03–5.30), and BA.2 antigen, CD4+ T cells (OR = 4.20, 95% CI 1.76–10.0). The SARS-CoV-2 reinfection patients showed no significant immune response against Wuhan and BA.2 antigens compared with the non-reinfection group.

Crude analysis of the cellular immune response and associated factors are shown in Table 5. CD8+ T-cell response against the Wuhan antigen was linked to alcohol consumption and blood group A, while inversely linked with blood group B. CD4+ T-cell response against the Wuhan antigen was inversely linked with physical exercise. CD8+ T-cell response against the BA.2 antigen was associated with age, number of SARS-CoV-2 vaccine doses received (three SARS-CoV-2 vaccine doses versus 0–2) and anti-N IgM, and inversely associated with the time elapsed since the last vaccine dose or infection. CD4+ T cell response was associated with BMI.

Adjusted analysis of the cellular immune responses and associated factors is shown in Table 6. CD8+ T cell response against the Wuhan antigen was significantly associated with alcohol consumption (OR= 2.18, 95% CI 1.20–4.33), number of SARS-CoV-2 vaccine doses received (OR = 1.85, 95% CI 1.13–3.03) and blood group A (OR = 2.61, 95% CI 1.37–4.96), and significantly inversely associated with blood group B (OR = 0.34, 95% CI 0.13–0.89). CD4+ T cell response against the Wuhan antigen was significantly inversely associated with physical exercise (OR = 0.44, 95% CI 0.21–0.99). CD8+ T cell response against the BA.2 antigen was significantly associated with age (OR = 1.03, 95% CI 1.01–1.05) and anti-N IgM (OR = 5.51, 95% CI 1.92–25.5), and significantly inversely associated with the time elapsed since the last vaccine dose or infection (OR = 0.89, 95% CI 0.81–0.99).

Spearman’s correlation tests between anti-S IgG levels and CD4+ and CD8+ T cell responses against the Wuhan and BA.2 antigens are shown in Table 7. The naïve group did not show a significant correlation between the level of anti-S IgG levels and CD4+ and CD8+ T cell responses against the two antigens. In contrast, SARS-CoV-2–infected patients showed positive correlations between anti-S IgG levels and CD4+ and CD8+ T cell response against Wuhan and BA.2, and the CD4+ T cell response was significant against the Wuhan antigen (rs = 0.198 *p* = 0.006), with marginal significance to the rest of the correlations. Patients with sequelae had significant positive correlation between anti-S IgG levels and CD8+ T cell responses against the Wuhan antigen (rs = 0.233 *p* = 0.042), and the non-sequelae group showed a significant correlation between anti-S IgG levels and CD4+ T cell responses against the Wuhan antigen (rs = 0.214 *p* = 0.030). SARS-CoV-2 patients with reinfection or no reinfection did not have significant correlations between anti-S IgG levels and CD4+ and CD8+ T cell responses against the Wuhan and BA.2 antigens. The whole of the sample showed a significant correlation between anti-S IgG levels and CD4+ T cell response against the Wuhan antigen (rs = 0.203 *p* = 0.002).

### Sensitivity Analysis

In the sensitivity analysis, a total of 175 participants (77.7%) presented with a positive cellular immune response ≥0.10% against at least one antigen, and 50 participants (22.2%) had a lower or no response at all. The cellular immune response of CD4+ T cells against Wuhan and BA.2 antigens was 46.8% and 50.7%, respectively. CD8+ T cell response against Wuhan and BA.2 was 49.3% and 47.6%, respectively. When comparing naïve and SARS-CoV-2–infected patients, the naïve group had a higher positive immune response ≥0.10% of 97% (35/36) versus 74.1% (140/189) (*p* = 0.015). In total, 145 (64.4%) were CD4+ T cell responders, and 147 (65.3%) participants were CD8+ T cell responders.

The adjusted sensitivity analysis comparing the cellular immune responses of CD4+ and CD8+ T cells among the study groups is shown in Table 8. The naïve group presented higher percentages of immune responses than SARS-CoV-2–infected patients, but the differences were not significant. Patients with sequelae had a higher immune response than the group without sequelae, and the percentage of CD4+ T cells against the BA.2 antigen presented significant difference (*p* = 0.05). CD4+ and CD8+ T cell responses against the two antigens had no significant difference when SARS-CoV-2 patients with reinfection were compared with no-reinfection group.

The adjusted analysis of the cellular immune responses and associated factors considering positivity as ≥ 0.10% are shown in Table 9. Significantly associated factors with the immune responses against Wuhan antigens were the number of SARS-CoV-2 vaccine doses received (CD8+ T cells), A and B blood groups (CD4+ T cells) and anti-S IgG (CD4+ T cells). For BA.2 antigens, significantly associated factors with the immune responses were the time elapsed since the last vaccine dose or infection, anti-N IgM (CD8+ T cells) and anti-S IgG (CD4+ T cells).

When comparing the sensitivity analysis and the first approach (Table 10), several factors maintained significance in both analyses: the time elapsed since the last vaccine dose or infection (CD8 + T cells against the BA.2 antigen), the number of SARS-CoV-2 vaccine doses received (CD8+ T cells against the Wuhan antigen) and anti-N IgM (CD8+ T cells against the BA.2 antigen). A and B blood groups were associated with a change of immune response from CD8 + T cells to CD4+ T cells against the Wuhan antigen. However, age, alcohol consumption and physical exercise lost significance in the sensitivity analysis.

## 4. Discussion

In our cohort, which showed a high percentage of three SARS-CoV-2 vaccine dose coverage, an elevated percentage of participants showed CD4+ and CD8+ T cell immune responses against Wuhan and BA.2 antigens. The naïve group had a cellular immune response comparable to that of the SARS-CoV-2–infected patients. SARS-CoV-2–infected patients with sequelae had a more significant immune response than those without sequelae. In SARS-CoV-2–infected patients, anti-S IgG levels showed a significant correlation with the immune responses of CD4+ and CD8+ T cells.

Anti-N IgM, blood group A and the number of SARS-CoV-2 vaccine doses received were associated with T cell responses. Inversely associated factors of immune responses included time elapsed since the last vaccine dose or infection and blood group B. Older age, alcohol consumption and physical exercise were associated with the immune response, but the sensitivity analysis did not reproduce these associations.

In our cohort, the percentage of CD4+ and CD8+ T cell responders was higher than that observed in other studies at the population level or in healthcare workers in cross-sectional [30,31] or cohort studies [13,32,33,34,35,36,37]. However, several tests to measure immune response were used with diverse SARS-CoV-2 variants and the follow-up was variable, but reached a minimum of six months.

Previous studies had detected CD4+ and CD8+ T cells after the first dose of mRNA SARS-CoV-2 vaccines [19,38,39,40,41] with a duration of six months and more [42]. Our results, 11 months after the last vaccine dose in the naïve group, are in line with these studies. In the SARS-CoV-2–infected patients, CD4+ and CD8+ T cell responses had been previously reported after eight months of the onset of the disease with an increase of CD4+ versus CD8+ [43]. Subsequently, a decline of both T cell responses had been described [44,45], more so in CD4+ than in CD8+ [46]. In addition, SARS-CoV-2–reactive CD4+ T cells had been detected in 40–60% of non-exposed individuals, possibly due to previously circulating coronaviruses [47].

The presence of CD4+ and CD8+ T cells may indicate a protection against COVID-19, considering that SARS-CoV-2 patients with SARS-CoV-2 vaccine have the so-called hybrid immunity (HI) (infection plus vaccination), and experience reduced severity and fewer hospitalizations upon reinfection [48]. HI supposes a more robust cellular immunity and increased SARS-CoV-2–neutralizing antibodies [49,50,51]. Primorac and co-authors [52] found less SARS-CoV-2 infection or reinfection with high levels of cellular immune responses after vaccination and/or previous SARS-CoV-2 infection. In a cohort study, Zens and co-authors [13] found that IFN-gamma–producing S-reactive–T-cells presented a significantly lower risk of SARS-CoV-2 infection or reinfection. In contrast, T cell response did not reduce breakthrough risk of SARS-CoV-2 infection in an open-label trial in Austria [53], in a Danish cohort study [35] nor in mRNA-vaccinated nursing home residents in Spain [54].

Cellular immune responses against SARS-CoV-2 may show differences in T cell populations considering quantity, localization and functionality with variations depending on epidemiological, virological, and immunological situations [43,55]. Likewise, SARS-CoV-2 protection may be dependent upon the different viral variants, time since the last infection, number of vaccine doses or inoculum size. In addition, some virus variants, such as Omicron, do not produce a T cell boost [56], and complementary determining regions for HI and non-HI individuals present distinct profiles which suggest improved immunity in HI individuals [57].

However, how the T cells protect against severe SARS-CoV-2–infection is not well demonstrated. Kent and co-authors [12] have indicated no association between SARS-CoV-2 infection and either T cell response measured in blood, considering that T cell response has not been measured in mucosal or tissues and there is not a standardized T cell assay for comparison with different studies. In addition, there are several tests to determine T cell response, including flow cytometry immunoassay for intracellular cytokine staining [24], activation induced marker assay [35], immunoadsorbent spot (FluroSpot) assay [58], enzyme-linked immunospot (ELISpot) assay and cytokine secretion assay [59,60]. These tests are difficult to implement at the population level due to their labor-intensiveness and low performance [13]. Other tests, such as interferon-γ release assay (QuantiFERON^®^ SARS-CoV-2 Test) [61], are being used, although the sensitivity is low [62,63].

The cellular immune responses had equivalent levels between the naïve group and SARS-CoV-2 patients and agrees with Camacho and co-authors, [30] who found no differences between vaccinated infected cases and vaccinated naïve participants in a cross–sectional study in general population of Valencia Community; with Paniskaki and co-authors [64] in a cohort of vaccinated naïve and vaccinated SARS-CoV-2 patients in Germany and with Gatti and co-authors in a comparison between SARS-CoV-2 convalescent and naïve vaccinated patients in Milan after 2 years of infection [65]. In addition, a study in a nursing home compared the cellular immune response of naïve residents with SARS-CoV-2 patients after a third dose of mRNA Comirnaty vaccine [66]; in naïve participants, after mRNA SARS-CoV-2 vaccine, a T cell response was found [67]. Furthermore, De Marcos and co-authors [37] found no difference in cellular immune response to the Omicron variant in a cohort of naïve or SARS-CoV-2–infected vaccinated health workers. In Brazil, Azamor and co-authors [68] found that 120 days after the second dose of ChAadOx1 nCoV-19, the percentages of CD4+ and CD8+ T cells were higher in a non-infected SARS-CoV-2 group compared with an infected SARS-CoV-2 group. Our results of cellular immune responses in the naïve group and the SARS-CoV-2 patients follow this thread. In contrast, Moore and co-authors [34] found higher T cell immune responses after six months of vaccination in a cohort of healthcare workers in England vaccinated with a prior SARS-CoV-2 infection than naïve vaccinated, suggesting that both immune responses from infection and/or vaccination will be similar over time. However, the immune response to the new variants of SARS-CoV-2 may present differently regarding protecting infected and naïve groups [69].

In our results, SARS-CoV-2 patients with sequelae had more CD4+ T cell immune responses against BA.2 and Wuhan antigens and CD8+ T cells against Wuhan antigen than SARS-CoV-2 patients without sequelae. This may suggest a more recent reinfection with BA.2, the variant predominant in our zone in 2022, and a persistent effect of the Wuhan variant. Some authors have found that patients with long COVID-19 present with altered CD4+ and CD8+ T cell populations that could be associated with viral persistence [70]. In long COVID-19 patients, an increase of CD4+ and CD8+ T cells secreting IFN-gamma [71] has been observed, and Cruz and co-authors [72] found increased levels of both CD4+ and CD8+ T cells in long COVID-19 patients with lung sequelae. In patients with post–COVID-19 syndrome, Acosta Ampudia and co-authors [73] found increased levels of CD8+ effector T cells and CD4+ effector memory T cells after 11 months of follow-up. Paniskaki and co-authors [74] found an intense SARS-CoV-2–reactive CD8+ T cell response in SARS-CoV-2 patients with sequelae.

However, other authors found lower cellular immune responses in severe SARS-CoV-2 patients, such as in Italy [75]. In addition, Wu and co-authors [76] found similar levels of CD4+ and CD8+ T cells in SARS-CoV-2 patients with or without pulmonary sequelae, although patients with pulmonary sequelae presented intense disparity in immunity with an increased proportion of natural killer T cells and a lower percentage of B cells. In contrast, other authors found an increment of CD8+ T cell percentages in post-acute SARS-CoV-2 patients with sequelae [77], related to the severity of the disease [32,33]. In addition, an increase of a T-cell–exhaustion marker (sTim-3) in a Norwegian cohort of hospitalized SARS-CoV-2 patients with 3 months follow-up after discharge could be associated with long-term outcomes after a severe disease [78]. Thus, an elevated heterogeneity in the immune response has been indicated considering the different clinical outcomes and the heterogeneity of CD4+ T cell responses [4,79].

Regarding factors associated with the cellular immune response estimated in our study, the number of SARS-CoV-2 vaccine doses received was associated with an increased T cell response, as it has been indicated in previous studies [9]. The number of SARS-CoV-2 mRNA vaccine doses increased the immune response in a cohort in the United States [80] and it was associated with a CD4+ T cell response in a cohort of Norwegian seniors [81]. Age was associated with a positive T cell response in line with the study of Costa and co-authors [36] in a cohort of healthcare workers in Italy. However, in nursing home residents, the rate and frequency of detectable SARS-CoV-2 IFN-γ–T-cell responses after vaccination was lower than in controls in Spain [54], and in England, residents of long-term care facilities presented with lower T cell response with higher age [82]. In Greece, in a cohort of healthcare workers, females had a higher T cell response [83]. In Ireland, Townsend and co-authors [84], in a cohort of COVID-19 patients and non-infected controls, reported that activated CD4+ and CD8+ T cell responses increased with age, but no association with sex was found. However, in a cohort study in Denmark, Dietz and co-authors [35] indicated a hypocellular immune response in males and those aged older than 75 years but without significant differences, and mRNA vaccines increased T cell response. In Brazil, Fernandes and co-authors [45] found heterogeneous T cell responsiveness decreased in males, older patients and non-hospitalized patients in a cohort of convalescents and non-exposed controls. In Italy, Sabetta and co-authors conducted a cohort study [85] and did not find an association of cellular immune response with age or sex. In Japan, Tani and co-authors [31] detected, after a booster mRNA vaccine, an increase in T cell response in participants aged ≤40 years and those with adverse reactions to the second or third vaccine dose.

In accordance with Costa and co-authors [36], who found that non-O blood groups were associated with increased T cell responses, blood group A had significantly higher CD8+ T-cell responses, but the B blood group had a significantly inverse response. In addition, Gil-Manso and co-authors [86] found that blood group O presented significantly lower frequencies of specific CD4+ T cell responses compared with non-O blood groups. However, no associations between blood groups and cellular immune responses have been reported in other studies [31].

The time elapsed since the last vaccine dose or the infection was inversely associated with a low cellular immune response and agrees with several studies in SARS-CoV-2 patients [44,45] and cohorts of healthcare workers in Italy [36] and Bulgaria [87]. Alcohol consumption was associated with the CD8+ T cell response. In contrast, Tani and co-authors [31] had not found this association in a cohort of mRNA vaccinated individuals. On the other hand, higher lymphocyte counts in people who consumed alcohol have been found [88]. Smoking was not associated with T cell responses, in line with Tani and co-authors [31]. However, Costa and co-authors [36] found that current smoking increased T cell responses. Physical exercise showed an inverse association with the CD4+ T cell response, suggesting low protection against SARS-CoV-2. This contrasts with an experimental clinical trial in Arizona, where exercise after SARS-CoV-2 vaccination was associated with robustly mobilized SARS-CoV-2–specific T cells, but only in SARS-CoV-2 patients, as non-infected participants presented reduced T cell responses [89]. In addition, Barni and co-authors [90] have indicated in a review study that exercise increases the amounts of CD4+, IL-6 and leukocytes. However, the effect of exercise increasing susceptibility to infection is a debated subject [91]. Like in other studies [81,92], no increase in T cell response was found in relation to BMI. Chronic disease prevalence was not associated with a T cell response, as it has been found in some studies [81] but in contrast with other studies where chronic diseases were associated with T cell responses [11,93]. Vitamin D levels had no effect on T cell response, despite its role as an immune regulator [94]. Association of anti-S IgG with T cell response has been indicated [43]. However, no correlation was found in a cohort of SARS-CoV-2 patients in Cambodia with a follow-up of nine months [95]. No significant difference of anti-S IgG levels between the groups was found, except that the SARS-CoV-2 non-reinfection group had higher anti-S IgG levels than the reinfection group, suggesting protection in the first group [96,97]. It is remarkable that anti-N IgM was associated with CD8+ T cell response against the BA.2 antigen, as this was the SARS-CoV-2 variant more frequent in our zone during 2022.

This study has several strengths: first, the follow-up of this cohort offers more knowledge of the dynamic of the SARS-CoV-2 pandemic; second, the participation rate of this cohort was over 60%; third, the nested case–control design in a cohort is useful to measure variables which required complex tests [98]; fourth, the measurement of variables was made before the nested case–control was carried out, which reduces information bias; fifth, a random sampling of the cohort participants was obtained to perform the nested case–control study; sixth, we used flow cytometry for intracellular cytokine staining to determine the cellular immune response, which has higher sensitivity compared with other tests [21]; finally, the results have been adjusted for potential confounding factors by multivariable logistic regression models.

Our study presents limitations: first, cellular immune memory has multiple components, and we are studied only SARS-CoV-2–S-reactive interferon-γ–producing CD4+ and CD8+ T cells as a measure of cellular immune response [99]; second, the use of flow cytometry for intracellular cytokine staining is not yet standardized [21]; third, just two SARS-CoV-2 variants, Wuhan and BA.2, were included in the study; fourth, only T cell interferon-γ production functionality was studied [26,100]; fifth, our sample size is more or less comparable with many studies of SARS-CoV-2 cellular immune response, but could lack power when comparisons inside groups are made; sixth, a measure of SARS-CoV-2 exposure could improve our results [9]; seventh, information on sequelae was obtained from the participants themselves; eighth, some SARS-CoV-2 reinfections may be misclassified considering the reinfection definition of the European Center Disease and Prevention [28]; ninth, most of the SARS-CoV-2 infections were mild, with few severe cases and hospitalizations, and it may be not well-represent the SARS-CoV-2 pandemic; tenth, COVID-19 is new and some potential factors may be not included in this study; eleventh, although the results are adjusted some residual bias may remain; finally, no SARS-CoV-2 variants of infected patients were obtained.

The analysis of sensitivity has produced a more detailed approximation of the immune response in our cohort. The results of the cellular immune response between the naïve group and SARS-CoV-2–infected patients were similar and significant differences between SARS-CoV-2 patients with sequelae versus patients without sequelae were maintained. The consistency of four associated factors suggests their importance in the dynamic of cellular immunity.

## 5. Conclusions

Cellular immune responses were elevated and comparable between naïve and SARS-CoV-2 groups, but higher in SARS-CoV-2 patients with sequelae. Several factors were associated with the T cell response, including the number of SARS-CoV-2 vaccine doses received. This study adds information on the cellular immune response that could be useful to the surveillance of the SARS-CoV-2 pandemic, and these findings contribute valuable insights into the nuanced immune landscape shaped by SARS-CoV-2 infection and vaccination.

## Figures and Tables

**Table 1:** Distribution of variables and comparison between the groups: naïve participants versus SARS-CoV-2–infected patients, SARS-CoV-2 patients with sequelae versus without sequelae, and SARS-CoV-2 patients with reinfection versus no reinfection.

Groups	Naïve	SARS-CoV-2–Infected	Sequelae ^1^		Reinfection ^2^	
	Controls n = 36	Cases n = 189	Cases Sequelae n = 77	Controls Non-Sequelae n = 103	Cases Reinfection n = 78	Controls No Reinfection n = 88
Variables						
Age (years) Median (Range) ^3^	51 (1–82) **	44 (20–70)	47 (19–71) **	42 (14–82)	45 (18–69)	44 (20–71)
Male (%)	16 (44.4)	71 (37.6)	27 (35.1)	42 (40.8)	30 (38.5)	31 (35.2)
Time (months) ^3,4^	11 (10–13)	11 (1–28)	11 (1–17)	11 (4–28)	11 (1–18)	11 (4–28)
Body mass index ^3^ (kg/m^2^) Median (Range)	25.7 (17.5–44.4)	26.2 (15–41.5)	26.4 (16–41.5)	26 (15.1–41.2)	25.4 (16–40.6)	26.4 (17.3–41.5)
Smoking (%)	16 (44.4) **	38 (28.2)	19 (25.0)	15 (14.6)	13 (16.7)	23 (26.4)
Alcohol consumption (%)	28 (77.8)	139 (73.9)	58 (75.3)	75 (73.5)	58 (75.3)	67 (76.1)
Physical exercise	18 (50.0)	112 (59.3)	40 (52.0)	66 (64.1)	49 (62.8)	50 (56.8)
Chronic Disease (%)	12 (33.3)	73 (38.6)	34 (44.2)	35 (33.0)	29 (37.2)	37 (40.1)
Vaccinated ^5^ (%)	36 (100)	183 (96.8)	77 (100)	97 (94.2)	74 (94.9)	87 (98.9)
mRNA ^6^ only (%)	25 (69.4)	138 (75.4)	57 (74.0)	74 (76.3)	54 (73.0)	68 (78.2)
mRNA+ others (%)	11 (30.6)	45 (24.6)	20 (26.0)	23 (23.7)	20 (27.0)	19 (21.8)
3 doses (%)	36 (100) **	126 (77.8)	53 (68.8) *	68 (66.0)	44 (56.4) *	63 (71.6)
2 doses (%)	0	53 (28.0)	24 (31.2)	25 (24.3)	26 (33.3)	24 (27.7)
1 doses (%)	0	4 (2.1)	0	4 (3.9)	4 (5.1)	0
0 doses (%)	0	6 (3.2)	0	6 (5.3)	4 (5.1)	1 (1.1)
Humoral immunity						
Anti-S IgG ^3,7^ AU/mL	1512 (214–5681)	1346 (115–5681)	1410 (372–5681)	1277 (115–5681)	1132 (115–5681) **	1653 (189–5681)
Anti-N IgG or IgM ^8^	0 (0)	116 (61.4)	44 (57.1)	68 (66.0)	56 (71.8) **	42 (47.7)
Anti-N IgM	0	25 (13.2)	9 (11.7)	16 (15.5)	14 (18.0)	8 (9.1)
Anti-N IgG	0	107 (56.6)	41 (53.3)	62 (60.2)	52 (66.7) **	39 (44.3)
Vit D ^3,9^ ng/mL	28.1 (11.9–64.5)	29.1 (12.5–70.8)	30.3 (13.9–70.8)	30.1 ± 9.4	30.2 ± 10.4	30.5 ± 9.3
Vit D ^9^ ≥30 ng/mL	13 (36.1)	86 (45.5)	41 (53.3)	43 (41.8)	34 (43.6)	44 (50.0)
ABO blood groups						
O	14 (58.9)	80 (42.3)	28 (36.4)	47 (45.6)	34 (43.6)	35 (40.0)
A	18 (50.0)	88 (46.6)	37 (48.1)	49 (47.6)	36 (46.2)	45 (51.1)
B	2 (5.6)	17 (8.0)	10 (13.0)	5 (4.9)	6 (7.7)	8 (9.1)
AB	2 (5.6)	2 (2.1)	2 (2.6)	2 (1.9)	2 (2.6)	0

^1^ Missing information n = 9. ^2^ No confirmation tests n = 23. ^3^ Median ranges 1%–95%. ^4^ Times since the last vaccine dose/infection. ^5^ One or more doses of any of the SARS-CoV-2 vaccines. ^6^ Messenger RNA vaccine. ^7^ Anti-spike IgG antibodies. ^8^ Total anti-nucleocapsid IgG or IgM antibodies. ^9^ Vitamin D. * *p*-value < 0.05. ** *p*-value < 0.01.

**Table 2:** Distribution of cellular immune response in naïve patients, SARS-CoV-2–infected patients, SARS-CoV-2 patients with sequelae or without sequelae, and SARS-CoV-2 patients with reinfection or no reinfection, measured by CD4+ and CD8+ T cell response against Wuhan and BA.2 antigens.

	Naïve Controls	Infected Patients Cases	Patients with Sequelae Cases	Patients with No Sequelae Controls	Patients with Reinfection Cases	Patients with No Reinfection Controls
T-cell response	Frequency median and range (%) ^1^					
CD8+ for BA.2 ^1^	0.11% (0–6.5%)	0.08% (0–13.3%)	0.09% (0–8.82%)	0.07% (0–13.3%)	0.08% (0–2.57%)	0.10% (0–13.3%)
CD4+ for BA.2 ^1^	0.13% (0–0.85%)	0.09% (0–2.37%)	0.11% (0–2.37%)	0.07% (0–1.67)	0.09% (0–2.37%)	0.10% (0–1.67%)
CD8+ for Wuhan ^1^	0.10% (0–3.56%)	0.09% (0–13.5%)	0.10% (0–3.25%)	0.08% (0–13.5%)	0.07% (0–6.90%)	0.11% (0–13.5%)
CD4+ for Wuhan ^1^	0.10% (0–1.05%)	0.08% (0–2.03%)	0.08% (0–1.15%)	0.08% (0–2.03%)	0.07% (0–1.47%)	0.09% (0–2.03%)
	Number of positives (%)					
CD8+ for BA.2 ^2^	28 (77.8%)	130 (68.8%)	58 (75.3%)	67 (65.0%)	53 (68.0%)	61 (69.3%)
CD4+ for BA.2 ^2^	31 (86.1%)	144 (76.2%)	67 (87.0%)	69 (67.0%)	60 (76.9%)	67 (76.1%)
CD8+ for Wuhan ^2^	29 (80.6%)	136 (72.0%)	61 (79.2%)	68 (66.0%)	52 (66.7%)	66 (75.0%)
CD4+ for Wuhan ^2^	32 (88.9%)	146 (77.3%)	69 (89.6%)	72 (69.9%)	59 (75.6%)	70 (79.6%)

^1^ Median (range) of CD8+ and CD4+ T cell response against BA.2 and Wuhan variants. ^2^ Percentage of number of positive CD8+ and CD4+ T cell responses against BA.2 and Wuhan variants.

**Table 3:** Crude analysis of cellular immune response comparisons between the study groups: naïve versus SARS-CoV-2–infected patients, SARS-CoV-2 patients with sequelae versus without sequelae, and SARS-CoV-2 patients with reinfection versus no reinfection by logistic regression. Odds ratio (OR) 95% Confidence Interval (CI).

	CD8+ for BA.2	CD4+ for BA.2	CD8+ for Wuhan	CD4+ for Wuhan
Groups	OR (95% CI)	OR (95% CI)	OR (95% CI)	OR (95% CI)
Infected patients Cases	0.63 (0.77–1.46)	0.52 (0.19–1.41)	0.62 (0.25–1.50)	0.42 (0.14–1.27)
Naïve Controls	1.00	1.00	1.00	1.00
Patients with sequelae Cases	1.64 (0.85–3.17)	3.20 (1.51–7.31)	1.96 (0.99–3.89)	3.71 (1.60–8.64)
Patients without sequelae Controls	1.00	1.00	1.00	1.00
Patients with reinfection Cases	0.93 (0.49–1.81)	1.01 (0.51–2.15)	0.67 (0.34–1.31)	0.80 (0.38–1.66)
Patients with no reinfection Controls	1.00	1.00	1.00	1.00

**Table 4:** Cellular immune response comparisons between the study groups: naïve versus SARS-CoV-2–infected patients, SARS-CoV-2 patients with sequelae versus without sequelae, and SARS-CoV-2 patients with reinfection versus no reinfection by logistic regression. Adjusted odds ratios (aOR). 95% Confidence interval (CI). Borriana COVID-19 cohort 2022.

Groups	CD8+ for BA.2	CD4+ for BA.2	CD8+ for Wuhan	CD4+ for Wuhan
	% positive	% positive	% positive	% positive
Infected Patients Cases	68.8%	76.2%	72.0%	77.3%
Naïve Controls	77.8%	86.1%	80.6%	88.9%
aOR ^1^ (96% CI)	0.81 (0.32–2.08)	0.39 (0.13–1.19)	1.01 (0.37–2.77)	0.45 (0.14–1.49)
*p*-value	0.668	0.097	0.982	0.191
Patients with sequelae Cases	75.3%	87.0%	79.2%	89.6%
Patients without sequelae Controls	65.1%	67.0%	66.0%	69.9%
aOR ^1^ (95% CI)	1.24 (0.59–2.62)	4.20 (1.76–10.0)	2.33 (1.03–5.30)	3.90 (1.50–9.52)
*p*-value	0.569	0.001	0.043	0.004
Patients with reinfection Cases	67.0%	76.0%	66.7%	75.6%
Patients with no reinfection Controls	69.3%	76.1	75.0%	79.6%
aOR ^1^ (95% CI)	0.94 (0.44–1.47)	0.80 (0.36–1.78)	0.84 (0.38–1.89)	0.80 (0.35–1.85)
*p*-value	0.860	0.584	0.659	0.602

^1^ Adjusted for age, sex, blood groups, body mass index, chronic disease, smoker, alcohol consumption, physical exercise, vaccine doses, time since the last vaccine dose/infection.

**Table 5:** Crude analysis of cellular immune response against Wuhan and BA.2 antigens and associated factors by logistic regression across the total sample. Odds ratio (OR) and 95% Confidence Interval (CI).

	CD8+ for BA.2	CD4+ for BA.2	CD8+ for Wuhan	CD4+ for Wuhan
Variables	OR 95% CI	OR 95% CI	OR 95% CI	OR 95% CI
Age (years)	1.03 (1.0–1.05)	1.01 (0.93–1.03)	1.01 (0.98–1.03)	1.01 (0.98–1.03)
Male	1.09 (0.60–1.96)	0.84 (0.44–1.58)	1.24 (0.67–2.29)	0.81 (0.42–1.57)
Time (months) ^1^	0.89 (0.81–0.98)	0.95 (0.86–1.05)	1.05 (0.95–1.15)	1.07 (0.97–1.18)
Body mass index (kg/m^2^)	1.02 (0.96–1.08)	1.08 (1.01–1.15)	1.04 (0.98–1.10)	1.02 (0.95–1.09)
Smoking	0.81 (0.49–1.81)	0.67 (0.33–1.36)	0.83 (0.42–1.63)	0.69 (0.34–1.41)
Alcohol consumption	0.99 (0.51–1.92)	1.04 (0.57–2.13)	1.98 (1.04–3.79)	1.04 (0.50–2.19)
Physical exercise	0.72 (0.41–1.31)	0.87 (0.45–1.65)	0.78 (0.13–1.43)	0.50 (0.25–0.99)
Chronic Disease	1.64 (0.89–3.02)	1.23 (0.63–2.39)	1.07 (0.57–1.97)	1.78 (0.88–3.60)
SARS-CoV-2 Vaccine				
mRNA homologous vaccine	1.39 (0.72–2.67)	0.96 (0.46–2.01)	1.05 (0.53–2.10)	0.80 (0.36–1.73)
3 doses versus 0, 1, or 2 doses	1.88 (1.02–3.48)	1.13 (0.57–2.26)	1.42 (0.75–2.69)	0.74 (0.35–1.57)
Number of vaccine doses	1.70 (1.12–2.60)	1.15 (0.73–1.82)	1.41 (0.92–2.15)	1.10 (0.68–1.38)
Humoral immunity				
Anti-S IgG (AU/mL)	1.00 (0.99–1.00)	1.00 (0.94–1.10)	1.00 (0.99–1.00)	1.01 (0.94–1.10)
Anti-N IgM or IgG	1.04 (0.54–1.85)	1.08 (0.58–2.03)	1.19 (0.66–2.15)	0.92 (0.48–1.75)
Anti-N IgM	5.54 (1.27–24.2)	1.16 (0.41–3.27)	2.04 (0.67–6.21)	2.07 (0.59–7.22)
Anti-N IgG	0.83 (0.47–1.48)	0.88 (0.47–1.65)	1.05 (0.58–1.90)	0.53 (0.44–1.59)
Vitamin D ng/mL	1.00 (0.97–1.03)	0.99 (0.96–1.02)	0.99 (0.96–1.03)	0.94 (0.97–1.03)
Vitamin D ≥30 ng/mL	0.88 (0.49–1.56)	0.90 (0.48–1.69)	0.95 (0.62–1.71)	1.20 (0.62–2.39)
ABO blood groups				
O	0.65 (0.36–1.15)	0.80 (0.43–1.51)	0.63 (0.35–1.15)	0.69 (0.36–1.32)
A	1.36 (0.76–2.42)	1.05 (0.56–1.99)	2.41 (1.29–4.49)	1.26 (0.66–2.41)
B	1.21 (0.42–3.49)	1.57 (0.44–5.64)	0.37 (0.14–0.95)	0.99 (0.31–3.13)
AB	2.16 (0.24–18.8)	1.44 (0.16–12.6)	0.72 (0.13–4.04)	NC ^2^

^1^ Time since the last vaccine dose or infection. ^2^ NC= non-calculable.

**Table 6:** Cellular immune response against Wuhan and BA.2 antigens and associated factors by logistic regression across the total sample. Adjusted odds ratio (aOR) and 95% Confidence Interval (CI).

	CD8+ for BA.2	CD4+ for BA.2	CD8 + for Wuhan	CD4 + for Wuhan
Variables	aOR 95% CI	aOR 95% CI	aOR 95% CI	aOR 95% CI
Age ^1^ (years)	1.03 (1.01–1.05)	1.01 (0.99–1.03)	1.01 (0.99–1.03)	1.01 (0.98–1.03)
Male ^2^	1.01 (0.53–1.91)	0.80 (0.41–1.56)	1.13 (0.59–2.18)	0.68 (0.26–2.86)
Time ^3^ (months)	0.89 (0.81–0.99)	0.96 (0.87–1.06)	1.07 (0.97–1.19)	1.0 (0.98–1.21)
Body mass index ^4^ (kg/m^2^)	0.99 (0.94–1.05)	1.07 (0.99–1.15)	1.02 (0.96–1.09)	1.01 (0.94–1.08)
Smoking ^5^	0.76 (0.38–1.51)	0.63 (0.31–1.30)	0.82 (0.40–1.72)	0.73 (0.34–1.57)
Alcohol consumption ^6^	1.18 (0.59–2.36)	1.15 (0.55–2.40)	2.18 (1.20–4.33)	1.03 (0.52–2.42)
Physical exercise ^7^	0.66 (0.36–1.22)	0.83 (0.44–1.60)	0.77 (0.41–1.41)	0.44 (0.21–0.99)
Chronic Disease ^8^	1.03 (0.52–2.07)	0.90 (0.43–1.90)	0.87 (0.42–1.76)	1.72 (0.78–3.79)
SARS-CoV-2 Vaccine				
mRNA homologous vaccine ^9^	1.55 (0.77–3.11)	1.04 (0.48–2.26)	0.92 (0.44–1.92)	0.82 (0.36–1.87)
3 doses versus ^9^ 0, 1, or 2 doses	1.24 (0.60–2.56)	0.87 (0.39–1.93)	1.93 (0.92–4.05)	0.74 (0.35–1.69)
Number of vaccine doses ^9^	1.34 (0.83–2.17)	0.99 (0.58–1.68)	1.85 (1.13–3.03)	1.19 (0.68–1.38)
Humoral immunity				
Anti-S IgG ^10^ (AU/mL)	1.00 (0.99–1.00)	1.00 (0.99–1.00)	1.00 (0.99–1.00)	1.01 (0.99–1.00)
Anti-N ^10^	1.42 (0.74–2.73)	1.31 (0.57–2.21)	1.06 (0.54–2.09)	0.93 (0.46–1.89)
Anti-N IgM ^10^	5.51 (1.92–25.5)	1.01 (0.35–2.96)	2.31 (0.71–7.48)	1.98 (0.54–7.28)
Anti-N IgG ^10^	1.13 (0.59–2.17)	0.89 (0.44–1.80)	0.92 (0.47–1.83)	0.85 (0.42–1.73)
Vitamin D ^11^ ng/mL	1.01 (0.97–1.04)	1.01 (0.97–1.04)	1.01 (0.97–1.04)	1.01 (0.98–1.05)
Vitamin D >29 ng/mL ^11^	1.07 (0.56–2.04)	1.20 (0.60–2.40)	0.95 (0.43–1.86)	1.30 (0.64–2.68)
ABO blood groups				
O ^12^	0.71 (0.39–1.24)	0.83 (0.44–1.58)	0.61 (0.33–1.12)	0.65 (0.34–1.26)
A ^12^	1.14 (0.68–2.25)	1.01 (0.54–1.92)	2.61 (1.37–4.96)	1.36 (0.70–2.64)
B ^12^	1.29 (0.43–3.84)	1.64 (0.45–5.89)	0.34 (0.13–0.89)	0.92 (0.29–2.66)
AB ^12^	1.72 (0.19–15.8)	1.37 (0.15–12.2)	0.68 (0.11–3.89)	NC ^13^

Adjusted for: ^1^ Sex, blood group and time since the last vaccine dose or infection; ^2^ age, blood groups and time since the last vaccine dose or infection; ^3^ age, sex and blood groups; ^4^ age, sex, blood groups, smoking, alcohol consumption, physical exercise and time since the last vaccine dose or infection; ^5^ age, sex, blood groups, alcohol consumption, physical exercise and time since the last vaccine dose or infection; ^6^ age, sex, blood groups, physical exercise and time since the last vaccine dose or infection; ^7^ age, sex, blood groups, alcohol consumption, time since the last vaccine dose or infection; ^8^ age, sex, body mass index, blood groups, alcohol consumption, physical exercise, smoking and time since the last vaccine dose or infection; ^9^ age, sex, blood groups, chronic disease and time since the last vaccine dose or infection; ^10^ age, sex, blood groups, chronic disease, body mass index, alcohol consumption, physical exercise, smoking and time since the last vaccine dose or infection; ^11^ age, sex, blood groups, chronic disease, body mass index, alcohol consumption, physical exercise, smoking and time since the last vaccine dose or infection; ^12^ age, sex, time since the last vaccine dose or infection. ^13^ Non-calculable.

**Table 7:** Spearman’s correlation tests between anti-S IgG levels and CD4+ and CD8+ T-cell response for naïve and SARS-CoV-2–infected groups. Spearman’s correlation coefficient (rs).

Group	CD8 + for BA.2	CD4+ for BA.2	CD8+ for Wuhan	CD4+ for Wuhan
	rs *p*-value	rs *p*-value	rs *p*-value	rs *p*-value
Naïve group	−0.04 0.812	0.014 0.934	−0.133 0.438	0.224 0.188
Infected patients	0.14 0.055	0.134 0.065	0.138 0.059	0.198 0.006
Patients with sequelae	0.182 0.112	0.116 0.317	0.233 0.042	0.184 0.108
Patients without sequelae	0.143 0.151	0.068 0.493	0.093 0.348	0.214 0.030
Patients with reinfection	0.121 0.293	0.142 0.216	0.129 0.280	0.218 0.055
Patients with no reinfection	0.129 0.232	0.163 0.130	0.112 0.300	0.157 0.144
Total sample	0.122 0.068	0.118 0.078	0.103 0.124	0.203 0.002

**Table 8:** Sensitivity analysis of cellular immune responses of CD4+ and CD8+ T cells with positivity ≥ 0.10%. Comparisons between the naïve group versus SARS-CoV-2–infected patients, SARS-CoV-2 patients with sequelae versus without sequelae, and SARS-CoV-2 patients with reinfection versus no reinfection by logistic regression. Adjusted odds ratios (aOR). 95% Confidence interval (CI).

Groups	CD8+ for BA.2	CD4+ for BA.2	CD8+ for Wuhan	CD4+ for Wuhan
	% positive	% positive	% positive	% positive
Infected Patients Cases	46.6%	48.7%	49.1%	45.5%
Naïve Controls	52.8%	61.1%	50.0%	53.8%
aOR ^1^ (96% CI)	0.74 (0.34–1.43)	0.55 (0.24–1.24)	1.16 (0.53–2.55)	0.73 (0.33–1.61)
*p*-value	0.438	0.150	0.717	0.436
Patients with sequelae Cases	49.4%	55.8%	50.7%	46.8.%
Patients without sequelae Controls	45.6%	41.8%	47.6%	44.7%
aOR (95% CI)	0.99 (0.52–1.92)	1.96 (1.00–3.85)	1.05 (0.55–2.02)	1.16 (0.60–2.26)
*p*-value	0.495	0.050	0.875	0.657
Patients with reinfection Cases	44.9%	47.4.0%	43.6%	43.6%
Patients with no reinfection Controls	51.1%	50.0%	51.1%	50.0%
aOR (95% CI)	0.69 (0.35–1.38)	0.81 (0.41–1.63)	0.82 (0.42–1.61)	0.78 (0.40–1.54)
*p*-value	0.292	0.565	0.568	0.471

^1^ Adjusted for age, sex, blood groups, body mass index, chronic disease, smoker, alcohol consumption, physical exercise, dose vaccine and time since the last vaccine dose or infection.

**Table 9:** Sensitivity analysis of cellular immune responses against Wuhan and BA.2 antigens and associated factors by logistic regression across the total sample. Adjusted odds ratio (aOR) and 95% Confidence Interval (CI).

	CD8+ for BA.2	CD4+ for BA.2	CD8+ for Wuhan	CD4+ for Wuhan
Variables	aOR (95% CI)	aOR (95% CI)	aOR (95% CI)	aOR (95% CI)
Age ^1^ (years)	1.01 (0.99–1.03)	1.01 (0.99–1.03)	0.99 (0.97–1.01)	0.98 (0.97–1.01)
Male ^2^	1.18 (0.67–2.08)	1.14 (0.65–1.99)	1.06 (0.11–1.86)	1.02 (0.59–1.80)
Time ^3^ (months)	0.90 (0.83–0.99)	1.01 (0.93–1.10)	0.98 (0.91–1.07)	1.06 (0.97–1.15)
Body mass index ^4^ (kg/m^2^)	0.99 (0.94–1.05)	0.96 (0.91–1.01)	0.99 (0.94–1.04)	0.96 (0.91–1.01)
Smoking ^5^	0.67 (0.35–1.26)	0.53 (0.28–1.01)	0.83 (0.45–1.57)	0.94 (0.50–1.76)
Alcohol consumption ^6^	1.06 (0.57–1.58)	1.53 (0.82–2.85)	1.60 (0.86–3.00)	0.90 (0.42–1.68)
Physical exercise ^7^	0.71 (0.41–1.23)	0.83 (0.48–1.43)	0.89 (0.42–1.53)	0.84 (0.49–1.43)
Chronic Disease ^8^	1.13 (0.62–2.07)	0.90 (0.43–1.90)	0.87 (0.42–1.76)	1.72 (0.78–3.79)
SARS-CoV-2 Vaccine				
mRNA homologous vaccine ^9^	1.38 (0.73–2.61)	1.33 (0.70–2.50)	1.20 (0.64–2.25)	0.87 (0.47–1.64)
3 doses versus ^9^ 0, 1, or 2 doses	0.96 (0.50–1.85)	0.74 (0.38–1.92)	1.75 (0.91–3.38)	0.87 (0.46–1.67)
Number of vaccine doses ^9^	1.16 (0.73–1.83)	1.11 (0.71–1.73)	1.72 (1.05–2.82)	1.18 (0.76–1.86)
Humoral immunity				
Anti-S IgG ^10^ (AU/mL)	1.01 (0.99–1.01)	1.01 (1.00–1.01)	1.00 (0.99–1.00)	1.01 (1.0–1.01)
Anti-N ^10^	1.16 (0.66–2.06)	1.63 (0.91–2.89)	0.92 (0.52–1.62)	1.15 (0.65–2.04)
Anti-N IgM ^10^	2.88 (1.11–7.43)	2.10 (0.84–5.27)	2.31 (0.85–5.31)	2.27 (0.93–5.57)
Anti-N IgG ^10^	0.88 (0.50–1.57)	1,24 (0.70–2.19)	0.83 (0.47–1.98)	1.08 (0.61–1.91)
Vitamin D ^11^ (ng/mL)	1.02 (0.99–1.06)	0.99 (0.96–1.02)	1.01 (0.97–1.04)	1.01 (0.98–1.05)
Vitamin D >29 ^11^ (ng/mL)	1.19 (0.67–2.12)	0.73 (0.41–1.30)	0.74 (0.42–1.86)	0.87 (0.49–1.55)
ABO blood groups				
O ^12^	0.76 (0.44–1.31)	0.77 (0.45–1.32)	0.90 (0.53–1.54)	0.76 (0.44–1.31)
A ^12^	1.62 (0.94–2.77)	1.37 (0.81–2.34)	1.50 (0.88–2.56)	1.72 (1.00–2.95)
B ^12^	0.66 (0.25–2.76)	1.64 (0.25–1.74)	0.45 (0.16–1.23)	0.26 (0.08–0.82)
AB ^12^	0.43 (0.07–2.52)	1.37 (0.32–10.26)	0.50 (0.09–2.83)	2.66 (0.46–15.21)

Adjusted for: ^1^ sex, blood groups and time last vaccine doses or infection; ^2^ age, blood groups and time since the last vaccine dose or infection; ^3^ age, sex and blood groups; ^4^ age, sex, blood groups, smoking, alcohol consumption, physical exercise and time since the last vaccine dose or infection; ^5^ age, sex, blood groups, alcohol consumption, physical exercise and time since the last vaccine dose or infection; ^6^ age, sex, blood groups, physical exercise and time since the last vaccine dose or infection; ^7^ age, sex, blood groups, alcohol consumption and time since the last vaccine dose or infection; ^8^ age, sex, body mass index, blood groups, alcohol consumption, physical exercise, smoking and time since the last vaccine dose or infection; ^9^ age, sex, blood groups, chronic disease and time since the last vaccine dose or infection; ^10^ age, sex, blood groups, chronic disease, body mass index, alcohol consumption, physical exercise, smoking and time since the last vaccine dose or infection; ^11^ age, sex, blood groups, chronic disease, body mass index, alcohol consumption, physical exercise, smoking and time since the last vaccine dose or infection; ^12^ age, sex and time since the last vaccine dose or infection.

**Table 10:** Associated factors of cellular immune responses of CD4+ and CD8+ T cells considering positivity >0.0% versus positivity ≥ 0.10%. Logistic regression analysis. Adjusted odds ratio (aOR) and 95% confidence interval (CI).

Variables	Positive Cellular Immune Response > 0.0%	Positive Cellular Immune Response ≥ 0.10%
	aOR 95% CI	aOR 95% CI
Age (years)	1.03 (1.01–1.05) CD8+ BA.2	NS ^1^
Time (months)	0.89 (0.81–0.99) CD8 + BA.2	0.90 (0.83–0.99) CD8 + BA.2
Alcohol consumption	2.18 (1.20–4.33) CD8+ Wuhan	NS ^1^
Physical exercise	0.44 (0.21–0.99) CD4 + Wuhan	NS ^1^
Number of vaccine doses	1.85 (1.13–3.03) CD8 + Wuhan	1.72 (1.05–2.82) CD8+ Wuhan
Anti-S IgG (AU/mL)	NS ^1^	1.01 (1.00–1.01) CD4 + BA.2; CD4 + Wuhan
Anti-N IgM	5.51 (1.92–25.5) CD8 + BA.2	2.88 (1.11–7.43) CD8 + BA.2
A blood group	2.41 (1.29–4.49) CD8 + Wuhan	1.72 (1.00–2.95) CD4 + Wuhan
B blood group	0.37 (0.14–0.95) CD8 + Wuhan	0.26 (0.08–0.82) CD4 + Wuhan

^1^ NS = Not significant.

## Data Availability

Data from this study can be consulted if the authors are requested.
